# Smooth Muscle Progenitor Cells Preserve the Erectile Function by Reducing Corporal Smooth Muscle Cell Apoptosis after Bilateral Cavernous Nerve Crush Injury in Rats

**DOI:** 10.1155/2019/8520523

**Published:** 2019-11-16

**Authors:** Yi-No Wu, Kuo-Chiang Chen, Chun-Hou Liao, Chien-Liang Liu, Han-Sun Chiang

**Affiliations:** ^1^School of Medicine, Fu Jen Catholic University, New Taipei City, Taiwan; ^2^Department of Urology, Cathay General Hospital, Taipei, Taiwan; ^3^Division of Urology, Department of Surgery, Cardinal Tien Hospital, New Taipei City, Taiwan; ^4^Division of Urology, Department of Surgery, Chi Mei Hospital, Tainan City, Taiwan; ^5^Graduate Institute of Biomedical and Pharmaceutical Science, Fu Jen Catholic University, New Taipei City, Taiwan; ^6^Department of Urology, Fu Jen Catholic University Hospital, New Taipei City, Taiwan

## Abstract

Radical prostatectomy causes erectile dysfunction (ED) and irreversible morphologic changes, including induction of endothelial and smooth muscle cell (SMC) apoptosis in the corpus cavernosum (CC). The injection of smooth muscle progenitor cells (SPCs) thickens the vascular intima and has demonstrated therapeutic benefit in cardiovascular disease animal. Herein, we investigated the effect of SPCs on the recovery of erectile function (EF) in rat models with bilateral cavernous nerve (CN) injury. Twenty-four male Sprague-Dawley rats were randomized into sham, vehicle only, or SPC treatment groups. Rats in the SPC treatment and vehicle groups were subjected to bilateral CN injury before intracavernosal injection. Intracavernosal injections of SPCs increased all EF parameters at day 28 after injury and simultaneously reduced apoptosis of the SMCs. Ultrastructural analysis revealed that SPCs maintained the integrity of the CC by preserving the structure of the adherens junctions. Tracking transplanted SPCs labeled with EdU showed that transplanted SPCs remained in the CC 28 days after treatment. Intracavernosal SPC injection restored EF after bilateral CN injury by reducing SMC apoptosis, which favored the maintenance of the structure of adherens junctions and regulated the stability of corporal vessels. These findings demonstrate the therapeutic potential of SPCs for treating ED in humans.

## 1. Background

In urology, postprostatectomy erectile dysfunction (ED) complication occurring after radical prostatectomy (RP) for earlier prostate cancer is a topic of most concern. Nerve injury may also be the main cause of erectile dysfunction after surgery [1, 2]. Epidemiology of ED after RP range is from 60% to 70%; even when nerve-sparing techniques are applied, ED rates range is still high from 30% to 87% [3]. This type of ED is most likely due to a multifactorial process, including intraoperative neurogenic and vasculogenic injury. For many years, ED after radical surgery was overcome by the implantation of a penile prosthesis [4]. Several treatment approaches are currently available for clinical treatment after radical prostatectomy. Pharmacotherapeutic options include oral treatment (phosphodiesterase 5 (PDE5) inhibitor) and intracavernosal injections (PGE-1). The PDE5 inhibitors showed benefit only in 54% of ED patients in one study [5]. Moreover, PDE5 inhibitors cannot improve the unassisted erectile function (EF) with RP [6]. There is still no progress in terms of effective agents for ED. Our previous study was mainly to find the advance methods to protect the cavernous nerve because a neurogenic component likely plays an important role in the pathogenesis of the ED [7–9]. However, in our recent study, we found that CN injury induces the most severe ED and nerve damage, which is followed by partial spontaneous recovery of EF and regeneration of the CN at day 28 after injury. Defects in the corporal SMCs were irreversible after CN injury. This suggests that the protection of the corpus SMCs from apoptosis may represent a more important treatment modality than nerve protection in future studies using the CN crush injury model [10]. Therefore, an alternative treatment is highly desirable to develop an effective, clinical feasible medication for ED patients after RP.

Several pathophysiological theories have been proposed to explain ED after CN injury, including nNOS-positive nerve fiber decrease, vascular damage, corporal cavernosum inflammatory, cavernosal smooth muscle hypoxia and smooth muscle apoptosis and fibrosis [11]. Some reports have stated that neuromodulatory treatments are possible options, such as use of immunophilin ligands, neurotrophins, growth factors, and stem cell therapy, to prevent the development of hypoxia-induced tissue damage and fibrosis. Currently, stem cell-based therapy has been a promising strategy for neuroprotection or tissue-protection after bilateral CN injury [12]. It is difficult to translate to clinical use because of adverse pharmacokinetics and because it is hard for cancer patients to use.

Bone marrow-derived progenitor cells (BMPCs), including endothelial progenitor cells (EPCs) and smooth muscle progenitor cells (SPCs), can be derived from the autologous peripheral blood, and they differentiate more easily into vascular cells during arterial remodeling than stem cells. EPCs are capable of circulating, proliferating, and differentiating into mature endothelial cells [13]. We have also previously reported that EPC treatment restored EF in a rat model of bilateral CN injury through recruitment of EPCs toward the dorsal artery and preservation of corporal SMCs. These findings support the therapeutic potential of progenitor cells for treating ED in humans [14]. SPCs can limit plaque development and promote changes in plaque composition toward a stable phenotype in mice [15]. SPC-released angiopoietin-1 can facilitate stabilization of endothelial cell networks and may be responsible for tightly orchestrating the complex process of neovascularization. In addition, the proliferative ability of SPCs *in vitro* is greater than that of EPCs [16]. Close cooperation between endothelial cells and SMCs is important for the regulation of vessel maturation and stability.

The effects of intracavernous injections of SPCs for the treatment of ED after CN injury have not been explored to date. Given that SPCs can enhance the efficiency of proangiogenic cell-based therapy [17], the present study aimed to evaluate the therapeutic potential of SPCs injections into the corpora cavernosa of rats with bilateral CN injury.

## 2. Methods

### 2.1. Experimental Animals

Twenty-five 10-week-old male Sprague-Dawley rats (weight, 350–450 g) were used in this study. All animals were supplied by BioLasco Taiwan Co., Ltd. (Taipei, Taiwan), and approved by the Fu Jen Catholic University Animal Care and Use Committee (IACUC approval no.: A10425). All the animal experiment methods were conducted in accordance with the guidelines and regulation established by Fu Jen Catholic University, Taiwan.

### 2.2. Isolation and Culture of Smooth Muscle Progenitor Cells

Ten-week-old male Sprague-Dawley rats were used to isolate the SPCs from femurs. The bone marrow was diluted with phosphate-buffered saline (PBS). Mononuclear cells were obtained from the bone marrow by density gradient centrifugation (Histopaque-1083; Sigma–Aldrich, St. Louis, MO, USA) at 400 ×g for 30 minutes and then at 300 ×g for 10 minutes. The cell pellet was then suspended in endothelial basal medium-2 (EBM-2; Lonza Clonetics, Walkersville, MD, USA) supplemented with PDGF-BB (50 ng/mL). After 2-3 days, angioblast-like cells and spindle-shaped cell outgrowths were observed. SPCs were defined by cell morphology and immunofluorescence staining. To determine the potential activity of SPCs, cells were labeled with a long-term tracking dye using the EdU Cell Labeling Kit (Invitrogen) at different time points (6, 24, and 48 hours) and concentrations (10, 50, and 100 *μ*M) and then harvested.

### 2.3. Experimental Design and Surgical Procedures

Cavernous nerve crushing animal model was established by a standardized procedure to generate a nerve injury as described previously [7–10]. The rats were randomly assigned to 3 groups (8 rats per group): sham, vehicle only, and SPCs-treated groups. For the surgical procedure, the rats were first anesthetized with an intraperitoneal injection of sodium pentobarbital (50 mg/kg). After a lower abdomen midline incision, major pelvic ganglions and the CNs were observed and isolated. All rats were undergoing bilateral CN injury except sham group. The crushing injury of the CNs was applied using a hemostat clamp (Roboz Surgical Instrument Co. Inc., Gaithersburg, USA) for 2 minutes before closing the abdomen.

### 2.4. Measurement of Erectile Responses

The measurement of erectile function was determined by electrical stimulation of the CNs at 4 weeks after surgery to generate erectile responses as described previously [7–10]. The CNs were isolated from prostate, and the crura of the penis were identified. A 24 G needle containing 50 U/mL of heparin solution was inserted into the right penile crus. The MP36 pressure transducer (Biopac Systems Inc., CA, USA) was used to measure the intracavernous pressure (ICP) which was recorded by BSL 3.7.3 software. Then, the CNs were stimulated using a bipolar stainless steel electrode and the monophasic pulses were generated by a DS3 constant current stimulator (AutoMate Scientific Inc., CA, USA). The stimulus parameters included a 7.5 mA amplitude, a 20 Hz frequency, and a 0.2-millisecond pulse width of 60-second duration. Erectile responses were expressed as intracavernous pressure (ICP). The maximal ICP, the change in ICP (∆ICP), the area under the ICP curve (AUC), and the ratio of change in ICP and mean arterial pressure (MAP; ∆ICP/MAP) were assessed as the parameters of erectile function.

### 2.5. Immunofluorescence Staining

To determine the content of nNOS-positive nerve fiber and smooth muscle content in the cavernous nerve and penile tissue, we performed double immunofluorescent labeling for cavernous related tissue with antibodies as described previously [9, 10]. The nerve and penile tissue were fixed with formalin for 24 hours (10% formaldehyde w/v) and were subsequently embedded in paraffin. In the deparaffinized procedure, the slide was put in xylene for 10 minutes and this is repeated twice for a total of 3 treatments, followed by hydration in graded alcohols. Blocking procedures were incubated for 1 hour in 10% goat serum/2% bovine serum albumin/0.2% Triton X-100 (Sigma–Aldrich, St. Louis, MO, USA) at room temperature. The tissues were subsequently incubated overnight at 4°C with the following primary antibodies: rabbit anti-nNOS (Santa Cruz Biotechnology, Santa Cruz, CA, USA), mouse anti-neuron-specific *β*-III tubulin, and anti-*α*-smooth muscle actin (Abcam, Cambridge, UK). Then, the tissues were incubated with a 1 : 400 dilution of secondary antibody conjugated to Alexa Fluor 488 or Texas Red (Invitrogen, Carlsbad, CA, USA) for 1 hour. For further analysis of nNOS and SMC content by fluorescence microscopy, the ratios of the area of nNOS-positive cells to the area of *β*-III tubulin-positive cells in nerve fibers and the *α*-smooth muscle actin area of the corpus cavernosum (CC) were calculated at 400× and 50× magnifications, respectively. All computerized histomorphometric analyses were performed using ImageJ software (National Institutes of Health, Bethesda, MD, USA).

### 2.6. Statistical Analysis

Data were expressed as the mean ± standard deviation. Statistical analysis for comparison of multiple treatment groups was performed with a one-way analysis of variance (ANOVA) and pairwise post hoc comparisons with the Scheffé test by using the SPSS Version 12.0 (SPSS Inc., Chicago, IL, USA) for Windows. The *p* < 0.05 was considered to be statistically significant.

## 3. Results

### 3.1. Cultivation and Characteristic of Smooth Muscle Progenitor Cells

**Figure 1:**
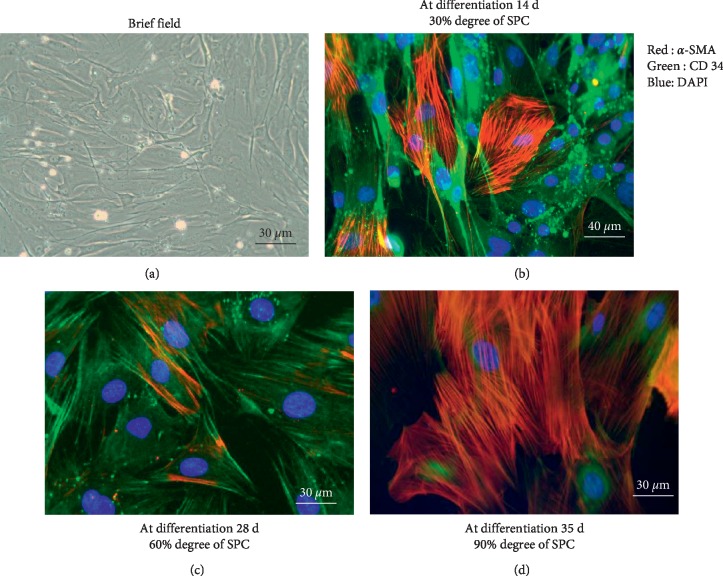
The characteristics of smooth muscle progenitor cells. (a) Outgrowth of smooth muscle progenitor cells (SPCs) by using the phase contrast microscope. (b–d) Immunofluorescence staining of SPCs. The cells were incubated with anti-*α*-SMA (red) and anti-CD34 (green) antibodies, respectively, and then complexed with Alex 594 and 488 secondary antibodies. Cell nuclei were counterstained with DAPI (blue).

Approximately 30% of the freshly isolated bone marrow mononuclear cells (BMMCs) adhered to culture plates after 3 days of culture with PDGF-BB-containing EGM-2-MV medium (Figure 1(a)). Two weeks after isolation and differentiation, 30% of outgrowth cells displayed specific morphological characteristics of SPCs as observed through fluorescence microscopy (Figure 1(b)). Thereafter, many SPC colonies were positive with CD34 and *α*-SMA antigen after differentiation at 4 weeks (about 60% SPC) (Figure 1(c)) and at 5 weeks (about 90% SPC) (Figure 1(d)).

### 3.2. Smooth Muscle Progenitor Cells Treatment Restores Erectile Function

**Figure 2:**
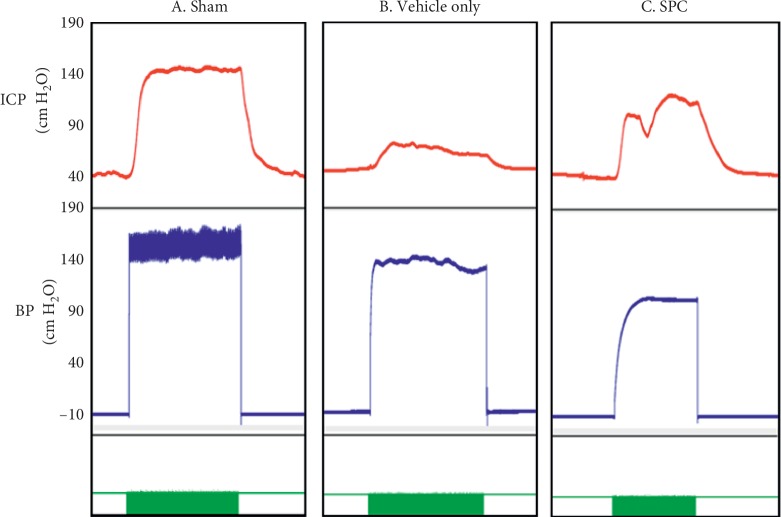
The measurement of intracavernosal pressure. Recordings of the intracavernosal pressure (ICP) and arterial blood pressure (BP) in the (A) sham, (B) vehicle only, and (C) smooth muscle progenitor cells- (SPCs-) injected mice. The *x*-axis represents time in seconds, and the green bar represents one electrical stimulus of 60 seconds. The *y*-axis represents the ICP and BP (top and bottom panels) in the experimental animals (*n* = 8).

Figure 2 presents the intracavernosal pressure (ICP) and the arterial blood pressure (BP) in the sham, vehicle only, and SPC-treated groups at 28 days after injury. The maximum ICP was significantly lower in the vehicle only treatment group compared with the sham group (56.19 ± 9.01 versus 141.72 ± 12.91 cm H_2_O, respectively; *p* < 0.001), which is consistent with ED. The maximum ICP in the SPC treatment group (127.85 ± 20.89 cm H_2_O; *p* < 0.001) was higher than that in the vehicle only group, but the difference between the SPC-treated and the sham groups was not significant. Similarly, other functional parameters of EF, such as ΔICP, area under curve (AUC), ΔICP/BP, and maximum ICP/BP, were significantly increased in the SPC-treated group (Table 1).

### 3.3. Intracavernous Injection of Smooth Muscle Progenitor Cells Can Prevent Atrophy of Corporal Smooth Muscle Cells

**Figure 3:**
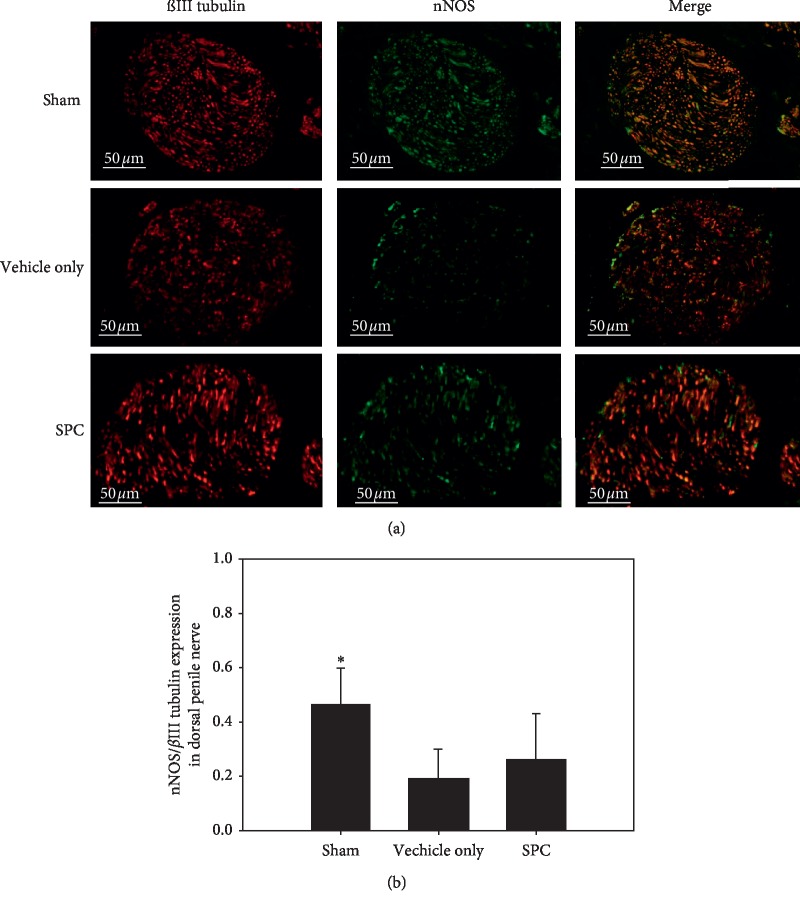
Immunofluorescence staining of nNOS in dorsal penile nerve. (a) Representative images of the dorsal penile nerve of each treatment group (original magnification: 400×). (b) Graph showing the quantification of nNOS-positive nerve fibers in the dorsal penile nerve, calculated as the area of nNOS-positive nerve fibers/*β*-III tubulin. ^*∗*^*p* < 0.05 versus vehicle only group.

**Figure 4:**
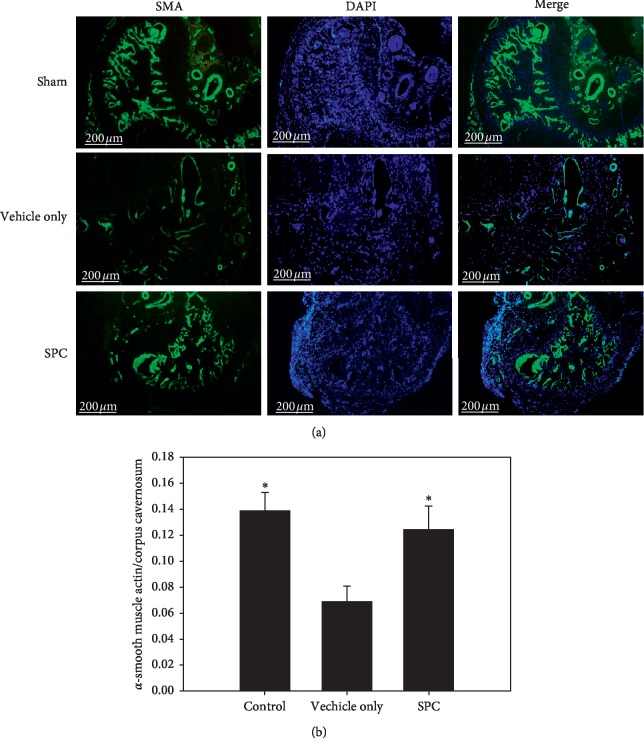
Immunofluorescence staining of *α*-SMA in smooth muscle cell of corpus cavernosum. Histological analyses of the corpus cavernosum (CC) 28 days after injury. (a) Representative fluorescence images of *α*-SMA-positive areas in the rat penile CC (smooth muscle, green; nuclei, blue) (original magnification: 50×). (b) Graph showing the smooth muscle cell (SMC) content in the CC quantified as the *α*-SMA-positive area/CC area. ^*∗*^*p* < 0.05 versus the vehicle group.

The nNOS-positive nerve fibers of the dorsal penile nerve were assessed by immunofluorescence staining.  Figure 3(a) depicts the immunostaining of the dorsal penile nerves for nNOS and *β*-III tubulin to quantify their nNOS content. Quantitative analysis showed that the ratio of the area of expression of nNOS/*β*-III tubulin was dramatically reduced in the vehicle only group compared with the sham group; however, there was no significant increase in the number of nNOS-positive nerve fibers after SPC treatment (Figure 3(b)). The corporal SMC content in the corpora cavernosa was evaluated by *α*-smooth muscle actin staining (Figure 4(a)). The SMC content within the corpora cavernosa was significantly lower in the vehicle only group compared with the sham and the SPC treatment groups (all *p* < 0.05; Figure 4(b)). These results indicate that SPC can prevent the atrophy of corporal SMCs.

Intracavernous injection of smooth muscle progenitor cells maintains the structure of adherens junctions of the smooth muscle in the corpus cavernosum.

**Figure 5:**
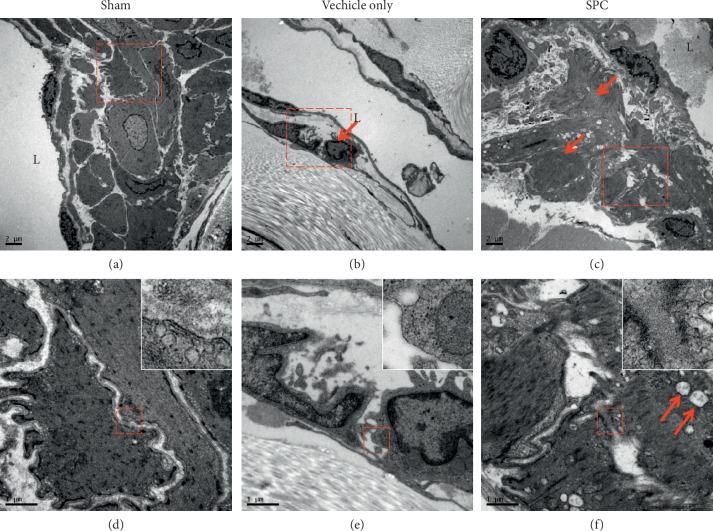
Ultrastructural analysis of the corpus cavernosum (CC) tissue. (a) Transmission electron microscopy (TEM) of the smooth muscle cell (SMC) of the CC near the sinusoid of the sham group. (b) A severe increase in extracellular matrix containing collagen was observed at vehicle only group. (c) Few apoptotic cells are observed in the smooth muscle area of CC after smooth muscle progenitor cells (SPC) treatment. (d) No apparent changes in the adherens junctions of the SMC were observed in the sham group. (e) A few fibroblasts are observed in the smooth muscle apoptotic site and loss of the adherens junctions in the SMC is shown. (f) Part of the adherens junctions between SMCs observed in the SPC group exhibits patterns of partial mitochondrial destruction (red arrow). The upper right square shows higher magnification in all groups.

Ultrastructural analysis of the CC tissue in the vehicle only and SPC-treated groups at 28 days after injury (Figure 5) was conducted by electron microscopy. Lower-magnification TEM images revealed healthy smooth muscle of the CC near the sinusoid in the sham groups (Figure 5(A)). Healthy smooth muscle was often located along the boundary of the endothelial cells and close to the collagen tissue. Higher-magnification micrographs suggested that the ultrastructure of the adherens junctions in the corporal tissue was intact and closely placed (Figure 5(D)). However, we observed abnormal structures and apoptosis of the SMCs in the vehicle only group (Figure 5(B)). In addition, loss of the adherens junctions in the SMC was also observed in the vehicle only group (Figure 5(E)). Few apoptotic cells were observed in the smooth muscle area of CC after SPC treatment (Figure 5(C)). Parts of the adherens junctions between the SMCs were observed in the SPC-treated group, but they exhibited patterns indicating partial destruction of mitochondria (Figure 5(F)).

### 3.4. Tracking of Smooth Muscle Progenitor Cells in the Corpus Cavernosum

**Figure 6:**
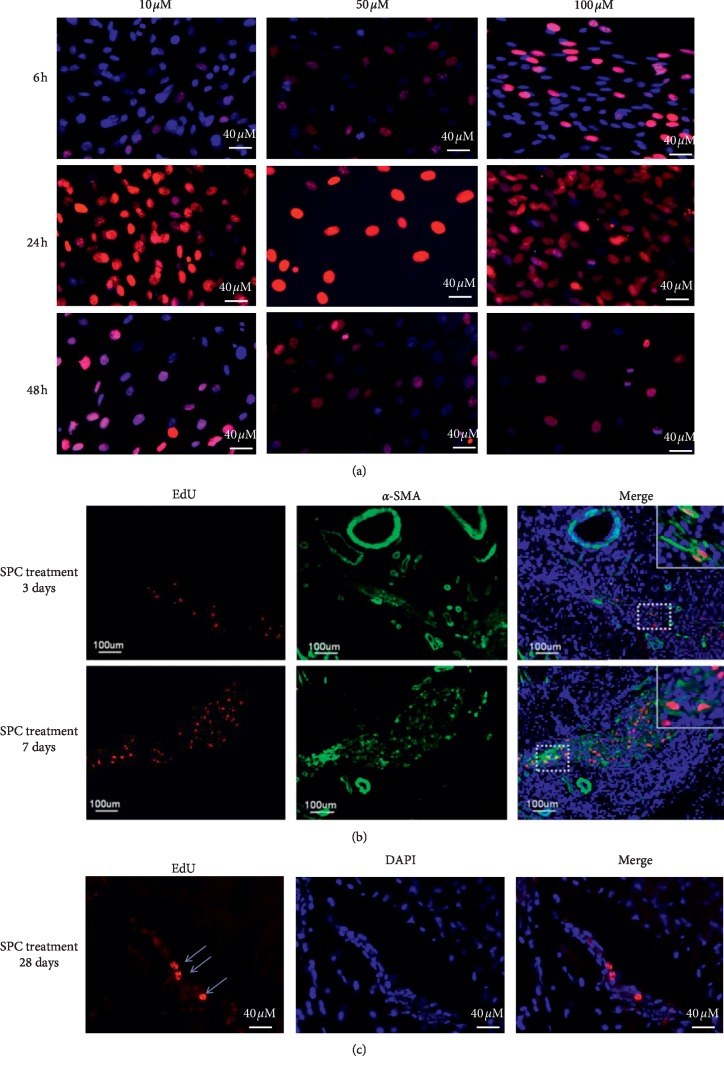
EdU labeling of smooth muscle progenitor cells (SPCs). (a) SPCs labeled with EdU at 10, 50, and 100 *μ*M and stained with Alexa-594 (red fluorescence) and DAPI (blue fluorescence) (200x magnification). Tracking corpus cavernosum-transplanted SPCs. (b, c) SPCs were labeled with EdU and injected into the corpus cavernosum tissue, which was then harvested at 3, 7, and 28 d and stained with Alexa-594 (red fluorescence) and DAPI (blue fluorescence). The Alexa-594 and DAPI stained images were digitally merged (scale bar = 40 *μ*m and 100 *μ*m).

To determine the potential activity of SPCs, cells were labeled with a long-term tracking dye using the EdU Cell Labeling Kit (Invitrogen) at different time points (6, 24, and 48 hours) and concentrations (10, 50, and 100 *μ*M). The optimal time point and concentration for EdU labeling occurred at 24 hours and at a concentration of 10 *μ*M, respectively (Figure 6(a)). The locations of EdU-positive (stained red) cells were determined near the vascular and nerve bundle at day 3 and day 7 (Figure 6(b)) and in sinusoid of corpus cavernosum at day 28 after SPC injection in the CC (Figure 6(c)).

## 4. Discussion

In this study, we report the beneficial tissue-protective effects of SPC intracavernosal injection therapy on restoring EF after CN crush injury. Unfortunately, the analysis of variance in nNOS expression revealed that there were no statistically significant differences between the SPC treatment group and the vehicle only group. However, the SPC treatment group showed significantly greater recovery of EF than the vehicle only group, as indicated by the higher levels of all EF parameters evaluated (including maximum ICP, ΔICP, AUC, and maximum ICP/BP) after treatment with SPCs (for all, *p* < 0.001). The beneficial tissue-protective effects of SPCs were attributed to an increased maintenance of the structure of the adherens junctions by SMCs due to the inhibition of cell apoptosis, suggesting their involvement in cavernosal tissue remodeling.

The components of penile erection include the cavernous nerve, the endothelium, and corporal smooth muscle. For optimal EF, accurate interactions among these three components are critical [14]. Following the CN crush in animals, nNOS expression in the penile nerves rapidly decreases and then begins to increase again over time [18]. The denervation of the penis induces significant apoptosis of corporal SMCs [19]. Corporal smooth muscle fibrosis may cause persistent ED even when other neurological functions recover after operation. In our recent study, we also found the damaged CN can lead to a partial spontaneous regeneration at 28 days after injury. Defects in the corporal SMCs were irreversible after CN crush injury [10]. Treatment with PDE5 inhibitors increases concentrations of cyclic guanosine monophosphate (cGMP); unfortunately, the composition of the corporal SMC will limit the effects of cGMP. Therefore, the main pathway of PDE5 inhibitor activity is disturbed in conditions of corporal SMC fibrosis. This may explain the low success rate of PDE5 inhibitors in treating post-RP ED. Thus, protecting the corporal SMCs from apoptosis using transplanted progenitor cells may be more important than nerve protection in patients undergoing nerve-sparing RP.

Adherens junctions are protein complexes that exist at cell-cell junctions in various cell types including epithelial, endothelial, and muscle cells, which play an essential role in intercellular tissue connections, mechanotransduction, and smooth muscle contraction [20, 21]. In this study, intracavernous injections of SPCs were able to sustain the structure of the adherens junctions of the SMCs, which play a vital role in smooth muscle contraction. In addition, we found that the smooth muscle in the contractile state was abundant around the sinusoid, which suggested that an increased presence of smooth muscle was able to maintain the contractile activity of the SMC intact and resulted in functional improvement. Rats that underwent injection of EdU-labeled SPCs for the purpose of tracking injected SPCs at 4 weeks after CN injury revealed that EdU-labeled SPCs were recruited to the damaged areas of the CC, implying that the therapeutic effect of SPCs was likely due to their mobilization and differentiation.

Currently, cell-based therapy has become a promising strategy for neuroprotection or tissue-protection after bilateral CN injury. The stem cell is capable of renewal and repair of tissue due to its capacity for division and differentiation [22]. Intracavernous injection of embryonic stem cells [23] and adult bone marrow-derived stem cells [24] has been reported to improve EF, but these studies have been limited due to clinical applicability and ethical concerns. Although adipose-derived stem cells (ADSCs) have found application in the regeneration of the CN in animal models and have been shown to be successful in the recovery of EF [25], stem cell differentiation does not easily occur during periods of repair [26]. The improvement of EF by ADSCs seems to be attributed to the paracrine action of cytokines rather than to the differentiation of ADSCs [27].

Proangiogenic cell-based therapy using autologous progenitors is a promising strategy for treating ischemic disease [28]. BMPCs, such as EPCs and SPCs, can be derived from peripheral blood and differentiate more easily into vascular cells during arterial remodeling than stem cells. EPCs are capable of circulating, proliferating, and differentiating into mature endothelial cells [13]. EPCs are required for endothelial repair and can participate in blood vessel formation [29]. The cavernous endothelium plays a crucial role in regulating the tone of the underlying smooth muscle and physiological penile erection [30]. We also reported previously that EPC treatment restored EF in a rat model of bilateral CN injury through the recruitment of EPCs toward the dorsal artery and preservation of SMCs in the CC. These findings corroborate the therapeutic potential of EPCs for treating ED in humans [14]. SPC-released angiopoietin-1 can facilitate stabilization of endothelial cell networks and may be more suitable to tightly orchestrate the complex process of neovascularization [16]. In addition, administration of SPCs can thicken the vascular intima and has demonstrated some therapeutic benefit for cardiovascular disease in animal models [31]. A tight cooperation between endothelial cells and SMCs is important to regulate vessel maturation and stability. Foubert et al. stated that SPCs may enhance the efficiency of treatment with EPCs [17]. More importantly, the proliferation ability of SPCs *in vitro* is higher than that of EPCs. Our study is the first report describing the use of SPCs to treat ED after CN injury and further elucidates the clinical application of SPCs in humans.

## 5. Conclusions

Treatment with SPCs restored EF in a rat model of bilateral CN crush injury through the recruitment of SPCs toward the sinuses in the corpora cavernosa, which sustained the structure of the adherens junctions of the SMC through a reduction of cellular apoptosis and increased stability of the corporal vessel. These findings provide support for the therapeutic potential of SPCs in treating ED in humans.

## Figures and Tables

**Table 1:** Intracavernous and mean arterial pressure measurements on cavernous nerve electrostimulation.

Groups	Maximum ICP (cm·H_2_O)	ΔICP (cm·H_2_O)	AUC	ΔICP/MAP	Maximum ICP/BP
Sham	141.72 ± 12.91^*∗*^	110.59 ± 12.79^*∗*^	4479.86 ± 948.83^*∗*^	0.68 ± 0.10^*∗*^	0.88 ± 0.0.12^*∗*^
Vehicle only	56.19 ± 9.01	33.00 ± 10.12	1059.71 ± 413.66	0.19 ± 0.06	0.33 ± 0.05
SPC	127.85 ± 20.89^*∗*^	100.85 ± 25.61^*∗*^	5193.72 ± 1585.04^*∗*^	0.64 ± 0.14^*∗*^	0.81 ± 0.12^*∗*^

SPC = smooth muscle progenitor cell; ICP = intracavernous pressure; ICP = Max ICP-Min ICP; MAP = mean arterial pressure; AUC = area under curve. ^*∗*^*P* < 0.001 versus vehicle only group.

## Data Availability

The data used to support the findings of this study are available from the corresponding author upon request.

## Abbreviations

ED: Erectile dysfunction
CC: Corpus cavernosum
SMC: Smooth muscle cell
SPCs: Smooth muscle progenitor cells
CN: Cavernous nerve
EPCs: Endothelial progenitor cells
BMPCs: Bone marrow-derived progenitor cells
ICP: Intracavernosal pressure
BP: Blood pressure
BMMCs: Bone marrow mononuclear cells
ADSCs: Adipose-derived stem cells.
